# Supplementary material for: Rice Genome Resequencing Reveals a Major Quantitative Trait Locus for Resistance to Bakanae Disease Caused by Fusarium fujikuroi
Source: Int J Mol Sci. 2019 May 27;20(10):2598. doi: 10.3390/ijms20102598 (PMC6566380; doi:10.3390/ijms20102598)
Supplement: Supplementary file 1 [file ijms-20-02598-s001.pdf]

Table S1. Distribution of DNA polymorphisms between ‘Junam’ and ‘Samgwang’ over chromosomes.

	SNP	InDel*	Sum
Chr1	8,371	897	9,268
Chr2	10,129	964	11,093
Chr3	7,615	600	8,215
Chr4	6,441	570	7,011
Chr5	2,479	293	2,772
Chr6	5,032	417	5,449
Chr7	15,620	1,532	17,152
Chr8	1,732	292	2,024
Chr9	6,527	475	7,002
Chr10	12,843	1,190	14,033
Chr11	51,628	3,282	54,910
Chr12	11,901	1,086	12,987
Total	140,318	11,598	151,916

* InDel is the abbreviation of insertion/deletion.

Table S2. Identification of QTLs for BD resistance using KASP markers

QTL name	chromosome	location (cM)	closest marker	QTL interval* (cM)	LOD	additive effect	dominance effect	R^2
qFfR9	9	29.2	*KJ09_024*	28.1-30.1	60.3	35.15	−1.27	0.59

* interval at 95% probability

Table S3. List of developed CAPS and dCAPS markers

No.	marker name	physical location		primer sequence		restriction enzyme	Marker type
		Chromosome number	distance from the top (bp)	forward	reverse		
1	9FC1	9	6,760,046	ACAACGAGAGGGATGGTGT	GGGTTCAACAAAGATCCAGA	PshBI	CAPS
2	9FC5	9	6,835,396	CACATGGTGAAAAGACATGG	TAGTGTGCAAAGCAGGACAA	PvuII	CAPS
3	9FC7	9	6,904,230	GGATGTTTTCTTTCTGATGACC	TAAGCCGTCATGTGGCACTA	XbaI	CAPS
4	9FC9	9	6,966,618	GCTTAACGCCGAGAGCTAGT	TCTAACAAAGTGGCATCACG	EcoRV	CAPS
5	9FC10	9	7,149,210	GTCCATTCCACCCTTACGTT	TTGATTGGGGGTTGAAGATA	EcoRV	CAPS
6	9FC13	9	7,182,057	TGCCATCATGAGTTGAAATG	AAAGGTCACCAGTGTGGAAA	PvuII	CAPS
7	9FC14	9	7,239,467	GGGGTAAAATGTGTTTGTGC	CTTGGGCTTTGTTTGTGAAC	PshBI	CAPS
8	9FC30	9	7,511,494	TGGAAGAAGAAAAGTAGGGAGA	ATAACGAAGCAGTCCCCAAA	HindIII	CAPS
9	JS9M6	9	14,581,727	AGAATGTAAGTCATTCTAGCAGATC	TGGTTAGCCTCATCGTAGAC	BglII	dCAPS

Table S4. Number of DNA polymorphisms in the genic region of genes located in the *qFfR9* region.

No.	gene ID	start*	end**	Orientation***	5' UTR	NS****	SY*****	intron	3'UTR	sum	gene description
1	Os09g0298100	7240336	7244713	+	0	0	1	0	0	1	Similar to predicted protein
2	Os09g0298200	7245434	7249813	-	0	0	0	0	0	0	Similar to Brittle 2
3	Os09g0298266	7245518	7247867	+	0	0	0	1	0	1	Hypothetical gene
4	Os09g0298332	7262920	7263701	+	4	5	3	4	1	17	Conserved hypothetical protein
5	Os09g0298400	7271228	7276768	+	1	2	0	18	0	21	WD40/YVTN repeat-like domain containing protein
6	Os09g0298500	7277255	7282211	-	0	1	1	2	0	4	Zinc finger RING/FYVE/PHD-type domain containing protein
7	Os09g0298700	7293013	7300542	-	0	4	7	14 (1)	0	25	Nucleotide-binding alpha-beta plait domain containing protein
8	Os09g0299000	7316075	7322207	+	0	0	0	18 (2)	7	25	Similar to CDPK substrate protein 1
9	Os09g0299200	7329560	7333289	+	0	1	0	4	0	5	Similar to MYB-CC type transfactor
10	Os09g0299300	7334523	7335240	+	0	0	0	4	2	6	Similar to predicted protein
11	Os09g0299400	7336243	7340325	-	0	1	4	9 (2)	0	14	Similar to TPK1

12	Os09g0299500	7347444	7351867	-	0	1	1	13	3	18	Similar to predicted protein
13	Os09g0300800	7470846	7483164	+	0	0	0	25	2	27	Protein of unknown function DUF247 plant family protein
14	Os09g0301800	7519040	7523188	+	10	2	1	0	1	14	Hypothetical conserved gene
15	Os09g0302233	7536715	7539761	+	0	0	0	30	0	30	Similar to Zinc knuckle domain-like
	Total				15	17	18	142	16	208	

* start position of the gene in reference genome

** end position of the gene in reference genome

*** orientation of the gene in reference genome

**** non-synonymous SNP

***** synonymous SNP

****** Among the total 208 DNA polymorphisms, 203 DNA polymorphisms were SNPs, and 5 DNA polymorphisms were InDels. All of the InDels were located in introns. The number of InDels were shown in parentheses.

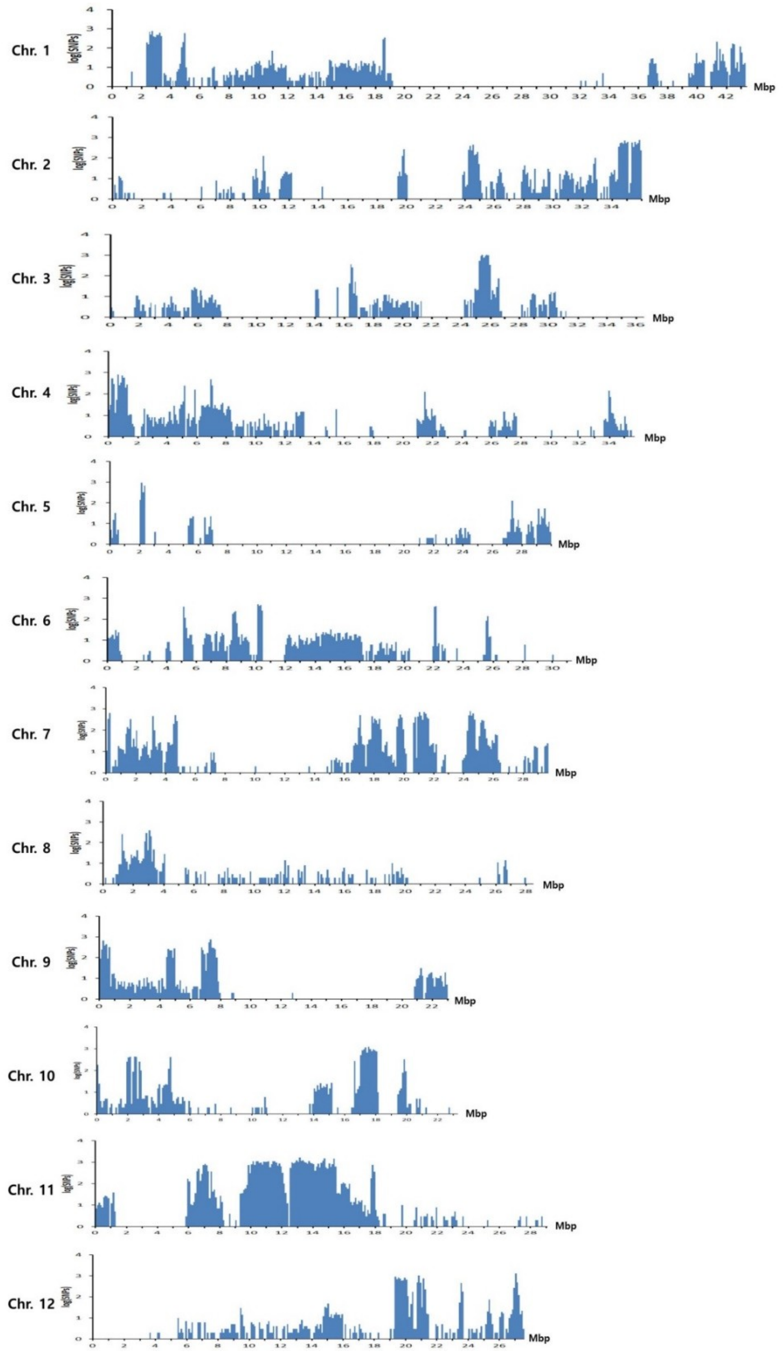

Figure S1 The distribution of DNA polymorphisms per 100 kb in the 12 chromosomes between the Junam and Samgwang rice varieties. X-axis represents the physical distance along each chromosome in mega base-pair (Mb) units; Y-axis indicates the common logarithm of the number of DNA polymorphisms.

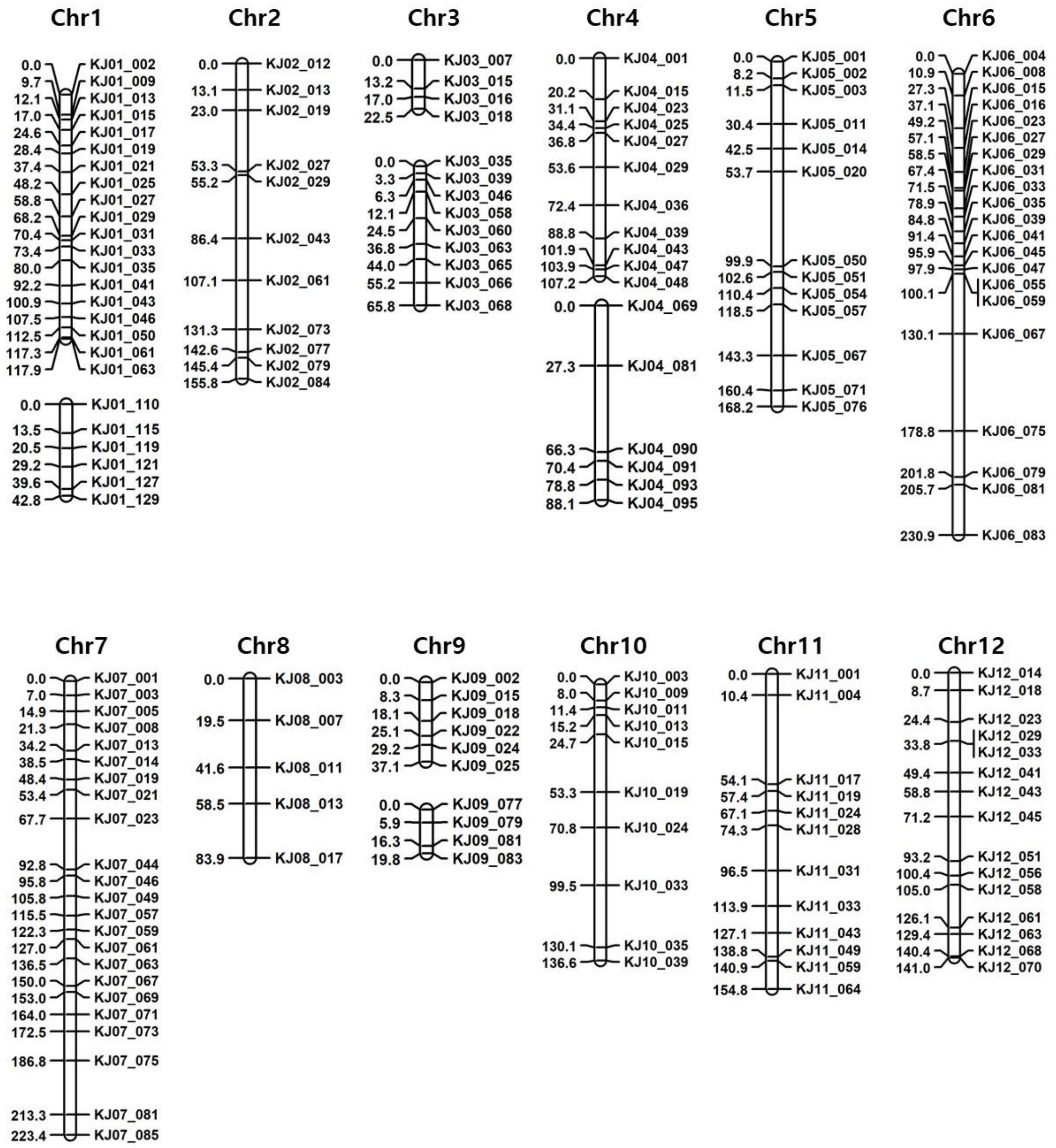

Figure S2 A genetic map constructed using KASP markers with the F2 plants derived from crossing of Junam and Samgwang varieties. The chromosome numbers are indicated on top of each chromosome, the name of each marker is indicated on the right-hand side of each chromosome, and the genetic distance of each marker from the first marker at the top of each chromosome is shown on the left-hand side. Genetic distances, measured as centimorgan or cM, were calculated using the Kosambi function. In each chromosome, the linkage groups were separated where the distance between adjacent markers exceeded 50 cM.

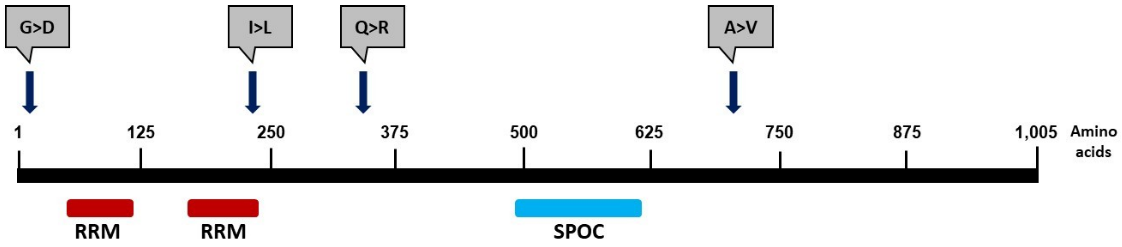

Figure S3 The domain structure of the deduced protein of Os09g0298700.

The RRM and SPOC domains are shown under the bar representing the length of the protein. RRM: RNA recognition domain. SPOC: Spen paralogue and orthologue C-terminal domain. The locations of amino acid changes between Junam and Samgwang are indicated by indigo arrows, and differences in amino acid were shown above the arrows.
